# A Flexible Mathematical Model Platform for Studying Branching Networks: Experimentally Validated Using the Model Actinomycete, *Streptomyces coelicolor*

**DOI:** 10.1371/journal.pone.0054316

**Published:** 2013-02-18

**Authors:** Leena Nieminen, Steven Webb, Margaret C. M. Smith, Paul A. Hoskisson

**Affiliations:** 1 Strathclyde Institute of Pharmacy and Biomedical Sciences, University of Strathclyde, Glasgow, United Kingdom; 2 Department of Mathematics and Statistics, University of Strathclyde, Glasgow, United Kingdom; 3 MRC Centre for Drug Safety Science, University of Liverpool, Liverpool, United Kingdom; 4 Department of Biology, University of York, York, United Kingdom; Vanderbilt University Medical Center, United States of America

## Abstract

Branching networks are ubiquitous in nature and their growth often responds to environmental cues dynamically. Using the antibiotic-producing soil bacterium *Streptomyces* as a model we have developed a flexible mathematical model platform for the study of branched biological networks. *Streptomyces* form large aggregates in liquid culture that can impair industrial antibiotic fermentations. Understanding the features of these could aid improvement of such processes. The model requires relatively few experimental values for parameterisation, yet delivers realistic simulations of *Streptomyces* pellet and is able to predict features, such as the density of hyphae, the number of growing tips and the location of antibiotic production within a pellet in response to pellet size and external nutrient supply. The model is scalable and will find utility in a range of branched biological networks such as angiogenesis, plant root growth and fungal hyphal networks.

## Introduction

Branched morphological networks are ubiquitous in biology and have received much attention in theoretical and experimental studies in a range of biological systems. Branched networks are highly scalable from bacterial hyphal structures in the μm to mm spatial range and minutes to hours timescales through to colonies of *Armillaria bulbosa* occupying 150,000 square metres over thousands of year timescales [1]. These scales represent a significant challenge to the modeling of such dynamic recursive structures, yet modeling these systems has been valuable in revealing many emergent properties of branched networks, yielding important details regarding angiogenesis in organs and tumors [2]–[6], transport networks in fungi [1], [7]–[9] and amoebae [2]–[6], [10] and the development of root systems in plants [11], [12]. Often however such models are system specific and lack flexibility. Thus an adaptable and flexible model platform that responds to external factors and can give an output in terms of the network heterogeneity that can be applied to many branched networks would be highly desirable.

The saprophytic soil bacterium *Streptomyces* is commercially exploited for the production of antibiotics, immunosuppressive, anticancer agents and other bioactive metabolites. *Streptomyces* are unusual bacteria in their growth form; they grow by apical extension of an individual hypha, achieving exponential growth by the addition of new hyphal tips through branching [13]. In their natural soil environment the hyphae access resources in a heterogeneous environment via this exploratory apical extension. In liquid culture, such as the conditions used commercially to produce bioactive metabolites, these apically extending and branching hyphae become entangled and form large hyphal aggregates, whose morphology can significantly affect the efficiency of industrial scale fermentations. These mycelial aggregates (or pellets) are physiologically heterogeneous – often metabolically active at the edges, yet nutrient starved and anoxic at the centre [14], [15]. It is known that the nutritional status of *Streptomyces* cells has a profound effect on the formation of secondary metabolites such as antibiotics [16], such that much of the biomass in an industrial fermentation may be non-productive in terms of the desired metabolite. Measuring the heterogeneity of these pellets is difficult experimentally and understanding the heterogeneity could have significant value in the manipulation of industrial organisms in terms of their morphology and their fermentation characteristics. This is particularly important through the application of molecular biology and genetics to alter the morphology of industrial strains for improved fermentation characteristics. Studies in the literature show that there is a critical pellet size for the production of the antibiotic erythromycin in *Saccharopolyspora erythrea* [17]. Similarly strain variants with altered branching frequency showed more desirable fermentation characteristics such as increased antibiotic production [18], [19]. Increasing fragmentation of strains through the manipulation of key cell division genes such as *ssgA* in *Streptomyces* has also shown that engineering of production strains offers great potential for improved fermentation efficiency and yield [18], [19]. The application of a robust modeling platform to this process should therefore yield key information regarding the production of secondary metabolites and how this can be manipulated.

Previously there has been a great deal of interest in modelling both fungal and bacterial hyphal growth [20]–[23]. Lattice-based modelling has been applied to fungal hyphal growth [9], [24] and recent modelling attempts of *Streptomyces* include a genome wide metabolic model reconstruction [25], a mechanistic based model of branching [26] and a morphological model of pellet growth [27], all of which have built upon earlier work modelling work focussed on filamentous fungi in particular [14], [28]–[30].

Here we present a discrete-continuum stochastic differential equation model platform that is applicable to many of the branched networks found in biology. The advantage of our model is that it uses relatively few parameters, of which most are directly derived from experimental data. We have validated and parameterised the model using experimental data and used the resulting model outputs to make biologically important inferences regarding the growth dynamics, physiological heterogeneity and antibiotic production in the industrially important bacterium *Streptomyces.* Using oxygen as a growth limiting substrate, we evaluate the influence of hyphal extension and branching on pellet formation and gain insight into the areas of the pellet producing antibiotics. This is important for applications where an optimal branching rate can influence production. The model can make predictions that are difficult or impossible to measure using experimental methods. We have used the model to gain quantitative insight into pellet growth characteristics, predicting quantities such as the hyphal growth unit (HGU) and the localisation of maximal branching. The flexibility of this modelling platform means that it can be applied to a wide range of branched biological networks such as plant root growth, angiogenesis and fungal mycelium.

## Results

### Mathematical Description and Experimental Validation of the Model

#### Elongation of network

To describe the elongation of an individual tip within the network, we constructed a 2D random walk model. The location of a tip over time is defined by an ordinary differential equation, where the i^th^ hyphal tip has a position (*x_i_* ∈ <$>\raster="rg1"<$>^2^) that varies over time (*t*) according to its velocity (*v_i_* ∈ <$>\raster="rg1"<$>^2^), namely;
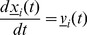
(1)


Equation 1 was solved using Euler's method. The direction of the tip movement is described by its velocity using a stochastic differential equation similar to that used by Stokes *et al.*, [2]. We further amend the model to include the average speed of extension (*v_avg_*) and normal velocity 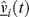 as shown in (2), where the velocity depends on the current value plus a random component which is unbiased and uncorrelated:
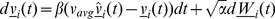
(2)where,



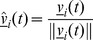
(3)and 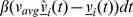 describes the tendency of the tip to move at the average elongation rate, and 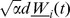 describes random fluctuations in tip speed and direction incorporating white noise into the model, where *β* is the drift coefficient and *α* is the diffusion coefficient.

The velocity of an individual tip is impossible to measure in liquid cultures and thus no speed distribution can be directly derived from experimental data. We assume that even when the hyphal network has grown to form a large pellet, the environment where the tips elongate and branches are formed, being at the extremities of the pellet, is still similar to the early hyphal growth (i.e. no growth limiting factors are minimal). Therefore, our assumption is that the velocity is unlikely to change dramatically during a pellet lifetime. The randomness incorporated in to the velocity is validated by model comparison against corresponding experimental observations of hyphal growth spread.

The model allows tip paths to cross over creating a 2D+ effect where we make the assumption that the overlapping tip paths are in different 3D planes. Therefore, the model is trying to capture 3D effects in 2D framework. This means that crowding is not explicitly included in the model, however it is intrinsically introduced via the growth limiting effects of an external substrate. The model has been rigorously validated and tested using laboratory studies to overcome any geometrical limitations of the 2D+ framework.

#### Branching

We mimic the natural process and model the branching of the network to occur behind the apically growing tip. The tip-to-branch distance is drawn from a cumulative probability distribution function with an average and standard deviation obtained from experimentally determined branching frequency distributions. The probability of branching increases with increasing apical length of an elongating tip. The length of a hypha (*L_i_*) is defined as:
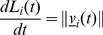
(4)


**Figure 1:**
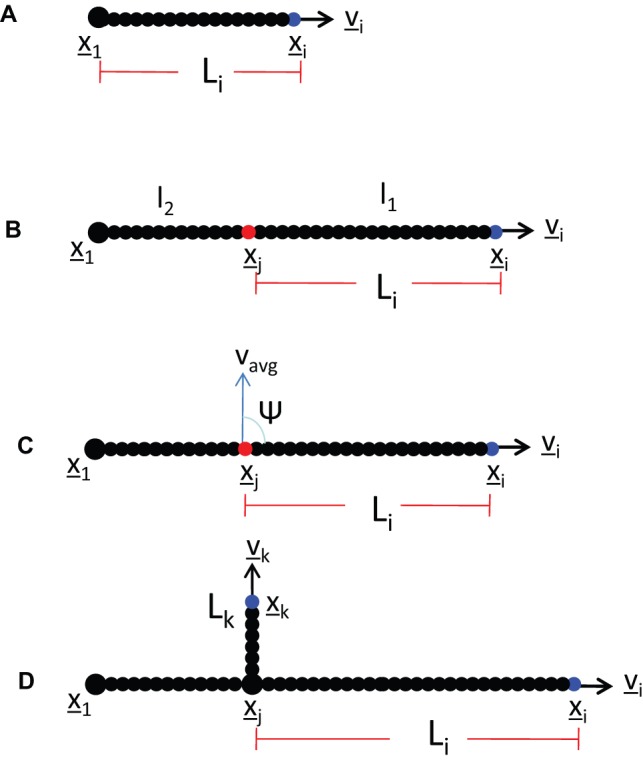
Branching events in the model. **A.** Hypha grows with velocity (*v_i_*) from its original position (*x_1_*). The total length of the hyphae is (*L_i_*). **B.** By a probability drawn from a normal distribution, a branching point *x_j_* is identified away from a starting point *x_1_* at an average inter-branch distance of *l_2_*, derived from experimentally observed values. **C.** A new hyphae emerges from the branching point position x_j_ with branching angle *φ* and average velocity *v_avg_*. The branching angle is taken from the parent hyphae using a bimodal probability distribution derived from experimental data. The average velocity (*v_avg_*) is measured from early hyphal growth experiments. **D.** Both the new and the parent hypha continue to elongate with their own velocities (*v_k_* and *v_i_* respectively). The length *L_i_* of the parent hyphae is now reset to be the distance between the latest branching point *x_j_* and its tip position *x_i_*, whereas the length of the new hyphae *L_k_* initiates from zero and increases as the new hyphae extends.

At each time step, a new position for each growing tip is calculated, the hyphal length updated and the probability of branching determined. The probability of branching is obtained from an experimentally derived cumulative distribution function (average l_1_+l_2_, standard deviation l_1_sd+l_2_sd) and is compared to a random number in the unit interval drawn from a uniform distribution. If the branching probability is higher than the randomly generated number, branching occurs and a branch point is created (note that we only allow branching to occur if the external substrate levels are high enough for active growth), therefore only growing cells can branch. The position of this new branching point is calculated from the start of the hyphae at an average interbranch distance (l_2_), which is experimentally derived. The branching angle (φ) is determined from a bimodal probability distribution derived from experimental data. The new tip first emerges from the branching point with an average velocity (v_avg_), measured from experimental data, and then continues to elongate on its own velocity independent from the parent hypha's velocity. The length of the new hypha increases from zero from the branching point and the length of the parent hypha is reset to be the distance between its tip position and the new branching point. The sub-apical branching event is depicted in **Fig. 1**. Information about the branching procedure is given in the Fig. 1 legend.

#### Validation of early network growth

At this stage, the model can be used to simulate early network growth where there are no rate limiting growth factors on the individual tips, e.g. such as substrate depletion. Out of total of eleven parameters used, only three cannot be measured directly from laboratory experiments in our model system, *Streptomyces* (see Table 1 for a full list of parameters). The first one of these parameters, the number of Brownian steps, was set to be 100 for a 4 h time interval. We found this sufficient for convergence of the Euler-Maruyama scheme used to numerically solve the stochastic differential equation (2). The most accurate values for the other two parameters, diffusion and drift coefficients, were determined using comparisons of simulations to equivalent experimental measurements of early *Streptomyces* hyphal growth experiments, where the external nutrient environment is constant and not limiting to growth.

**Table 1:** Parameters used for modelling early hyphal growth.

Parameter	Symbol	Value
Simulation time interval	T	4 h
Number of Brownian steps	_	100
Diffusion coefficient	*α*	10
Drift coefficient	*β*	10
Average apical length	*l_1_*	28.5 μm
Average interbranch length	*l_2_*	7.3 μm
Standard deviation of apical length	*l_1_sd*	8.5 μm
Standard deviation of interbranch length	*l_2_sd*	3.9 μm
Average branching angle	*φ*	±84.0 deg
Standard deviation of branching angle	*φ_sd_*	23.0 deg
Average hyphal velocity	*v_avg_*	6.3 μm/h

**Figure 2:**
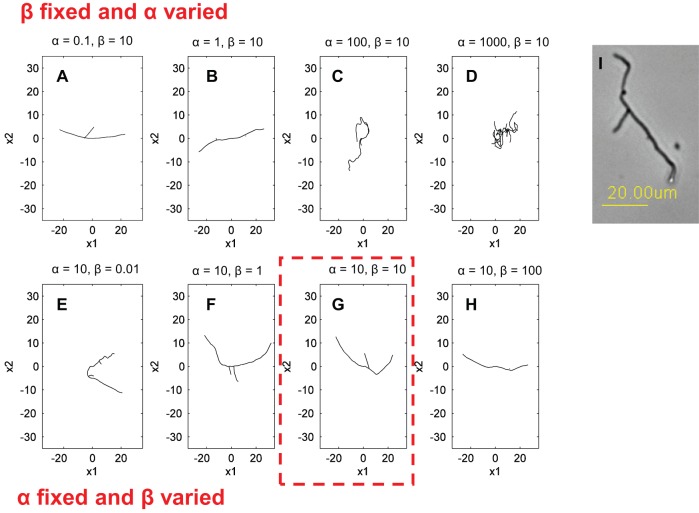
Simulation results for early network growth using different diffusion and drift coefficient values. **A–D.** Diffusion coefficient α varying from 0.1–1000, drift coefficient β is constant at 10. **E–H.** Drift coefficient β ranging from 0.01–100 when diffusion coefficient α stays constant 10. The remaining parameters are as per **Table** **1**. **I**. A phase contrast image of *S. coelicolor* early hyphal growth for comparison. The dashed box shows the simulation results for α and β values used in further simulations.

**Figure 3:**
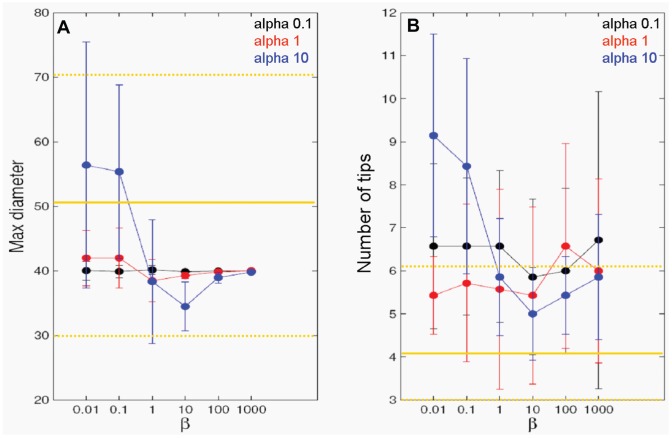
Numerical validation of early network growth. **A.** Comparison of maximum pellet diameter between the model and experimental data (n = 41). **B.** Comparison of number of tips between the model and experimental data (n = 44). The simulation results are presented as average with error bars showing the minimum to maximum values. The results from laboratory experiment are shown as yellow lines for mean (continuous line) and standard deviation (dashed line).

The simulation results for different diffusion α and drift β coefficient values are shown in **Fig. 2**, where subplots A– show the diffusion coefficient α varying from 0.1–000, when the drift coefficient β is taken to be constant. The bottom row subplots E– illustrate the difference between the drift coefficient values when they range from 0.01–100, whilst the diffusion coefficient stays constant. For experimental validation purposes the following conclusion can be drawn – the greater the diffusion coefficient the larger the random noise whereas with a large drift coefficient the resistance to random fluctuation is higher. Comparison of these simulations to microscopy images of early hyphal growth (Fig. 2 I), allows us to estimate the most appropriate alpha and beta values for this model organism. We confirmed this morphological observation by numerical calibration to the maximum pellet diameter and the number of tips in the **α** and **β** simulations to the corresponding experimental results. Simulations were run for values **α** (0.1–0) and **β** (0.01–000) (**Fig. 3**) and a comparison is made for each **α** and **β** value to the hyphal growth network shown in Fig. 2. From the comparison of the maximum pellet diameter it can be seen that the low **α** and high **β** values reduce the variation between the minimum and maximum values. To keep the minimum and maximum values within the scope of experimental standard deviations and still maintain as high a random variation as possible, only the **α** values of 1 and 10 and respective **β** values of 0.01–10 and 0.01–10 are taken forward. When comparing the effect **α** and **β** have to the number of the tips in the simulations, it is shown that with the value 10 for both **α** and **β** simulation output is closest to the average from the biological data, and with the variation between simulations staying within acceptable observed biological variation. From hereon in, we take α = β = 10 (indicated by the dashed box in **Fig. 2**).

### External environment

*Streptomyces* is grown in liquid cultures for the production of antibiotics, where the growing hyphae form dense pellets. Despite continuous mixing in these cultures nutrient and oxygen gradients can be generated inside pellets due to consumption, diffusion and mass transfer constraints, cell lysis and degradation processes [31]. We model a concentration of external substrate, 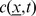 (mmol/l) where 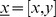, using a reaction-diffusion equation where the diffusion term is described by Fick's law [32]:
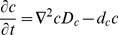
(5)where *D_c_* is the diffusion coefficient (μm^2^/h) and *d_c_* is the rate of consumption (h^−1^). For our experiments and simulations, the growth rate of the hyphae (hours) defines the time scale of interest. Compared to this time scale, over the length scale of a hyphal pellet the diffusion rate is fast and the consumption (*d_c_*) rate is also fast (order of seconds). We make use of these differences and assume a quasi-steady substrate concentration, to give the leading order expression:
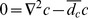
(6)where 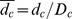. To solve this equation we subdivide the 2-D domain into a (NxN) regular square grid and discretize the laplacian using finite differences to give N^2^ coupled algebraic equations. Unless otherwise stated, we take N = 200 and a square domain of size 200×200 μm^2^.

We set the boundary conditions as being fixed to the substrate concentration that is detected in media in the absence of any cells. Initially a homogenous concentration is assumed throughout the grid. The tip paths are related to the underlying grid using a least squares interpolation. The hyphal consumption rate (*d_c_*) is then taken to be a function of hyphal occupancy density in the presence of cells. The density can then be calculated by interpolating each of the hyphal branches to the underlying square grid and counting the number of hyphae in different metabolic states in each grid square.

### Cell metabolism

We incorporate four different metabolic states of the hyphal aggregate into the model. We assume that as the external substrate concentration depletes due to diffusion limitation and cellular consumption, the hyphae switch from an active growth state (tip elongation and branching) to secondary metabolism state (the antibiotic producing state) as substrate limits growth [16]. As the substrate concentration depletes further the hyphae either die directly, or go through a state where only maintenance energy requirements are met, the cell is therefore alive but not growing or making antibiotic. From this state the hyphae are still able to recover active growth or antibiotic production states depending on the fluctuating external substrate concentration levels, or will then die. The consumption of substrate is assumed to decrease as the metabolism of hyphae change from actively growing to antibiotic producing and further to the maintenance state. Once the hypha dies, no substrate is consumed and it is not possible for the hypha to recover to previous metabolic states.

With the above assumptions, the substrate consumption rate in each square grid cell (i,j), i,j = 1…N is defined as
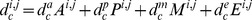
(7)where *A^i,j^*, *P^i,j^*, *M^i,j^* and *E^i,j^* are the numbers of actively growing, antibiotic producing, maintenance energy only and dead hyphae, respectively, in the grid cell (i,j). The parameters 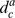, 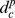, 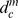 and 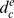 are taken to be the consumption rates for different metabolic states of hyphae. Note that 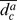, 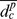, 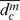 and 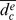 denote the corresponding rescaled (by D_c_) consumption rates. Note that 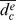 is zero for the simulations presented in this paper, however, if chemical oxygen demand for cell degradation processes are taken into account, this parameter can be adjusted accordingly.

### Validation of the model using oxygen as an external substrate

In our simulations, we use oxygen as an external substrate. We calculate the rescaled consumption rate (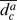) by taking into account the single cell dry weight, volume of a grid voxel (8 μm^3^ with the typical N and domain values indicated above, allowing the 2D+ effect of overlapping tip paths, as described above), the external oxygen concentration (*c*), the oxygen consumption rate (*d_c_*) and the oxygen diffusion coefficient (*D_c_*; Table 2). Since the mass of an average cell of *Streptomyces* is not known, mainly since the cell dimensions in filamentous organisms are hard to define [33], we make assumptions of the cell dry mass based on the *Esherichia coli* cell dry weight. To calculate the hypothetical cell volume for *Streptomyces coelicolor*, we exploit the fact that a single nucleus is associated with 1.9 μm hyphal length in vegetative hyphae and a hyphal diameter is known to vary between 0.5–1 μm [13]. Therefore, the *Streptomyces* single cell dimensions are assumed to be cylindrical with length of 1.9 μm and diameter of 1 μm. Then by assuming that an *E. coli* cell of the same volume weighs the same as a *Streptomyces* ‘cell’, we calculate a *Streptomyces* cell dry weight utilizing published *E. coli* cell dry weight measurements [34].

**Table 2:** Typical parameters used for external oxygen concentration and diffusion.

Parameter	Symbol	Value
O_2_ diffusion coefficient^1^	D	9.216×10^6^ μm^2^/h
O_2_ concentration (in absence of cells)	c	0.1975 mmol/l
O_2_ consumption rate^2^ – active growth	*d−_c_^a^*	1×10^−5^ μm^−2^
O_2_ consumption rate – antibiotic production	*d−_c_^p^*	70% of *d−_c_^a^*
O_2_ consumption rate – maintenance requirements only	*d−_c_^m^*	50% of *d−_c_^a^*
O_2_ consumption rate – dead	*d−_c_^e^*	0

1 Calculated using [45].

2 Consumption rate rescaled by diffusion rate.

The consumption rate of oxygen is estimated to be around 6.5 mmol g^−1^ h^−1^ for actively growing hyphae [35]. According to Melzoch *et al.* [35] continuous culture studies, at this rate, *Streptomyces coelicolor* M145 does not produce antibiotics, yet it is actively growing. By applying the above values to the rescaled oxygen consumption rate calculations (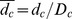), we are able to come to a value of ca 1×10^−6^ μm^−2^. It turns out, however, that this estimate produces a very dense pellet that is fully metabolically active with the core of the pellet consisting of antibiotic producing hyphae and no hyphae with only maintenance requirements. Our live/dead staining data suggest that the core of the pellet is likely to be inactive. Therefore simulations using the rescaled consumption rate of 1×10^−5^ μm^−2^ gave the most realistic comparison between our simulations and the experimentally data. We found this difference in the model parameter acceptable since the single cell dry weight is based on the above assumptions, and some of the data used in the calculations are derived from 3-dimensional studies (continuous culture studies), yet our model only takes into account 2-dimensional growth.

We estimated the varying levels of external oxygen concentration needed to change the metabolic states of the hypha based on the work of Melzoch *et al.* [35]. We made the assumptions that at 90–100% of initial oxygen concentration, all hyphae are assumed to be actively growing. When oxygen levels drop due to metabolic consumption, the probability of hyphae switching from an actively growing state to an antibiotic producing state increases. When the external oxygen levels are between 50–60% of the initial concentration, all the hypha are assumed to produce antibiotics. Antibiotic production stops when less than 40% of the initial oxygen concentration is present and the cells are only able to stay alive, but are non-growing between 15–46% oxygen. The cells die when less than 15% of the initial oxygen concentration level is available. It is noted that these parameters can be difficult to establish. We therefore performed a parameter sensitivity analysis on these parameters (omitted for brevity) and found no qualitative difference in the results.

### Pellet development

**Figure 4:**
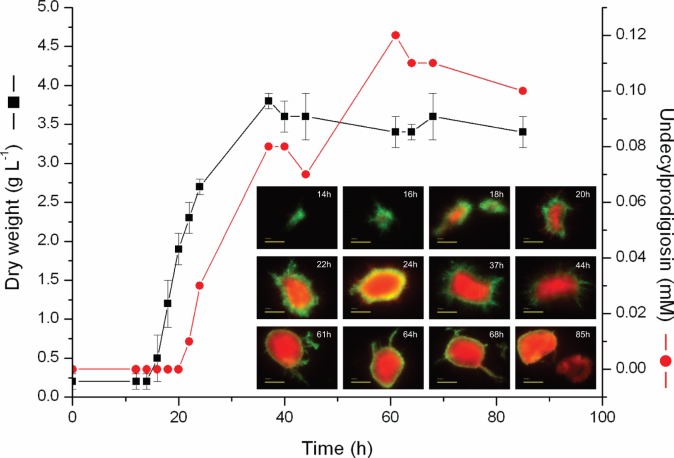
One generation of pellet development where BacLight live/dead staining highlights the cell heterogeneity within a pellet. As pellets grow in size, the red area spread from the core of the pellet to eventually covering the whole pellet. Green fluorescence (SYTO9) is associated with live cells; Red fluorescence propidium iodide (PI) stains dead cells. Bar  = 100 μm. Bacterial growth was monitored by dry weight weight (black squares). Antibiotic production is shown as undecylprodigiosin concentration (red circles). The error bars illustrate the standard deviation (n = 3).

*Streptomyces* grow by forming multigenomic, apically extending filamentous hyphae. Growth is initiated from a single spore and as growth proceeds in liquid cultures, the individual filaments get tangled together forming hyphal clumps and eventually dense pellets. Due to substrate diffusion limitations, the cells within a pellet are assumed to be heterogeneous in their metabolism. To illustrate this cell heterogeneity in a developing pellet, a *Streptomyces* growth curve is shown in **Fig. 4**, where growth is monitored by dry cell mass and cell pellets are stained using BacLight live/dead staining. Green areas correspond to the fluorescent dye, SYTO9 that stains cells with an intact membrane potential (Live cells). Red areas show hyphae that are stained with propidium iodide (PI), indicating cells with impaired membrane potential that are likely to be dead or at the very least metabolically inactive. Initial pellet development appears fully green implying that all the cells are live and active. Once the pellets develop further, the red areas appear first at the core of the pellet and eventually spread over the whole pellet, indicating a decrease in membrane potential and cell death. Some red staining hyphae are present throughout growth, likely representing natural variance in the system. Antibiotic production (the cell associated, red pigmented antibiotic, undecylprodigiosin [16]) was observed at 22–24 h onwards (**Fig. 4**). The cultures were largely comprised of dead cells from 61 hrs onwards and at the end of the growth curve, only the hyphal fragments, released from the edges of old pellets, remained active.

### Automated image analysis

**Figure 5:**
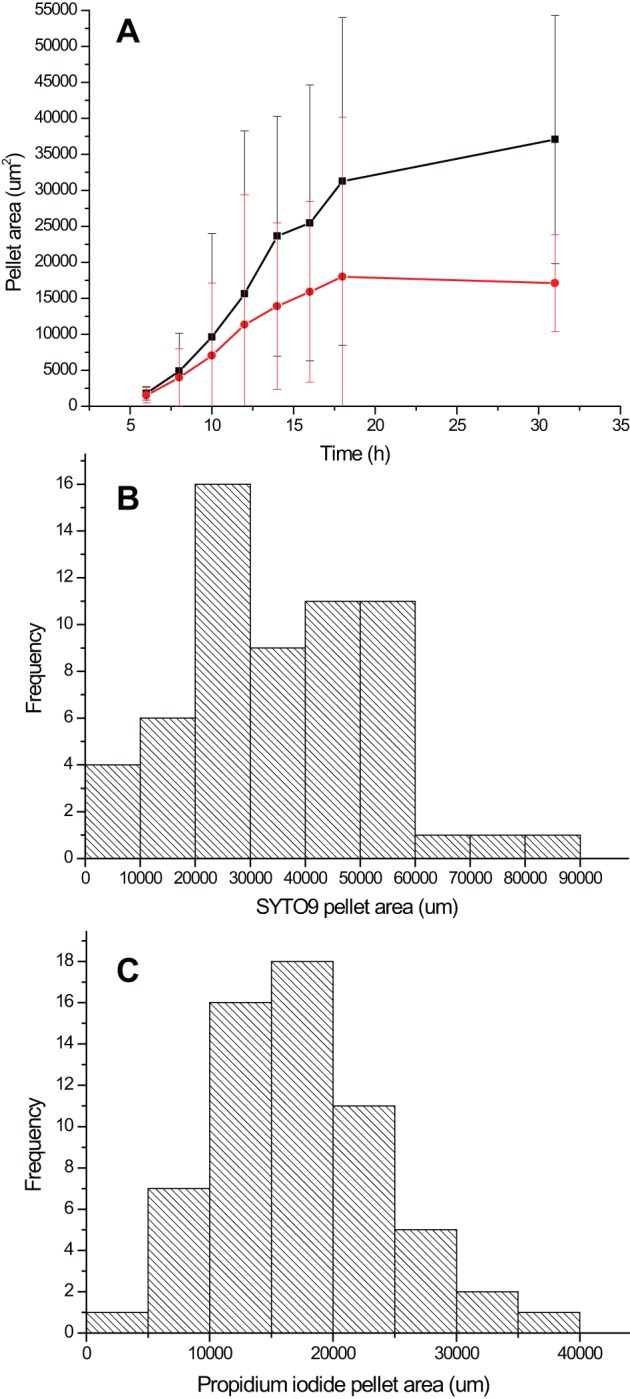
Results from automated image analysis of *Streptomyces coelicolor* pellets using live/dead staining. **A.** The mean area of SYTO9 (black squares) and PI (red diamonds) stained pellets over time. The total number of images included in the analysis was 892. The error bars correspond to the standard deviation of the data. At each time point, between 28 to 71 pellets were measured. Time scale adjusted to the modeling time line by deducting a 6 h germination period from the start of the growth curve. **B.** A frequency histogram of SYTO9 pellet areas at the end of the exponentially growing phase. The total number of pellets measured was 60. C. A frequency histogram at the end of the exponentially growing phase of pellet areas stained with PI. The number of pellets included was 61.

To gain insight into the pellet development, automated image analysis was performed on BacLight stained fluorescence images. During the rapid growing phase, the pellet area increased in size in both SYTO9 and PI stained hyphal parts (**Fig. 5**). At the end of the rapid growing phase (31 h), both the SYTO9 and PI pellet areas had relatively high coefficient variation (CV) of 46% and 39% respectively. From the frequency histograms presented in **Fig. 5**, it was observed that only few pellets out of ca 60 measurements contributed to this large distribution.

The average maximum pellet diameter was 270 μm for the SYTO9 fluorescence image analysis at the end of the exponential growth phase. By comparing manual measurements between SYTO9 fluorescence and phase-contrast images, it was seen that the average maximum pellet diameter in SYTO9 dyed images were 65.6% (CV 20.5) and 75.8% (CV 8.1) of the average maximum pellet diameter in phase-contrast images for time points 6–12 h and 14–31 h after germination respectively (Table 3). Consequently, the average maximum pellet diameter measured from SYTO9 fluorescence images gives results that are 35% and 24% smaller than the actual pellet for 6–12 h and 14–31 h after germination respectively. If the average value of 270 μm is corrected to represent the actual maximum pellet diameter, then the average maximum pellet diameter would be around 356 μm. The difference observed in fluorescence measurements and the phase contrast images of pellet development may be explained by the density of the pellets, such that the fluorescence from the pellet centre is higher than the fluorescence from the individual tips resulting in reduced detection of individual tips.

**Table 3:** The ratio of maximum pellet diameter in SYTO9 fluorescence images compared to phase-contrast images (manual measurements).

Time after germination^1^ (h)	Ratio of max pellet diameter (%)	n
6	67.2	31
8	64.9	53
10	66.3	10
12	63.8	10
14	74.4	10
16	76.6	10
18	75.4	10
31	76.7	10

1 Germination time 6 h.

### Comparison of Simulations to Experiments

**Figure 6:**
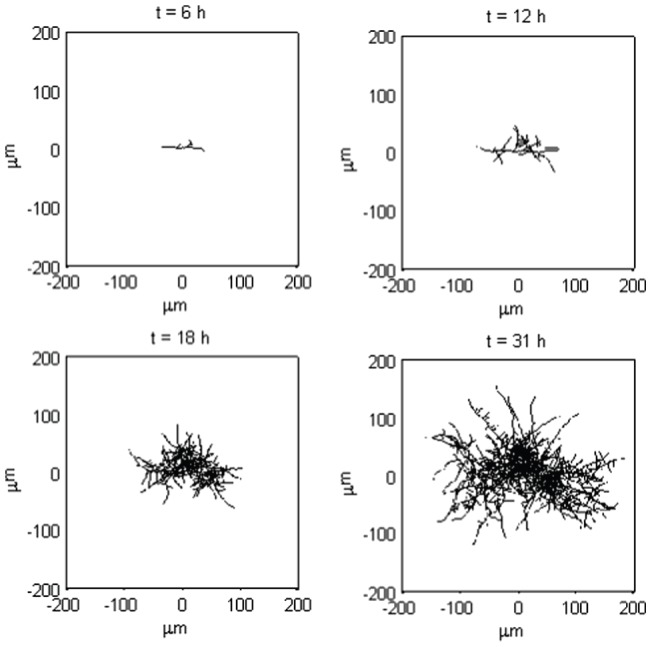
Example model simulation of network growth from a single cell to a dense network. The time points shown are 6 h, 12 h, 18 h and 31 h after germination. Details of the simulation procedure is given in the main text. Initially, two hyphal tips are considered to emerge from the origin *(x,y) = (0,0),* both with initial velocities *v_avg_* and random initial orientations.

We model the hyphal growth from a single cell to pellet formation and show an example of the simulated hyphal morphologies at 6 h, 12 h, 18 h and 31 h after germination (**Fig. 6**). Since no time lag is incorporated into the model for spore germination, the time shown is from the emergence of a germ tube, which in experiments was following approximately 6 hours of incubation. The simulation is clearly representative of the experimentally observed features. For example, when comparing pellet size (measured as the maximum pellet diameter) the pellet area and pellet perimeter, at the end of the simulation (31 h), it was noted that the simulations very accurately represent the biological variation observed at the end of rapid growth in the automated image analysis (**Table** **4**). The corrected value for the average maximum pellet diameter is 356 μm, and the corresponding value from the simulations is 380 μm; Therefore, the model correlates very accurately to the experimental data when comparing pellet diameters. The experimental data for pellet area gives a mode of between 20,000–30,000 and an average of 37,000. If this average is corrected to represent the phase contrast images of pellets (+24%) then it rescales to 46,000. This is still smaller than the 60,000 estimated from the simulations, but is likely to reflect additional complexities in the experiment such as additional growth limiting factors. It is noted that the antibiotic producing cells emerge in the simulations at around 16 h after germination. This corresponds to the timing observed for the production of undecylprodigiosin in our growth curve experiments.

**Table 4:** Comparison of simulation results to experimentally determined values.

Measurement^1^	Model simulation	Experiment (from min to max)^2^
max pellet diameter (μm)	380	90–50
pellet area (μm^2^)	60,000	250–90,000^3^
pellet perimeter (μm)	1250	260–3600

1 Measured at 31 h after germination.

2 Measured from SYTO9 fluorescence images, n = 60.

3 See histogram in Fig. 5.

### Exploiting Model Simulations

#### Cell heterogeneity and oxygen limitation

**Figure 7:**
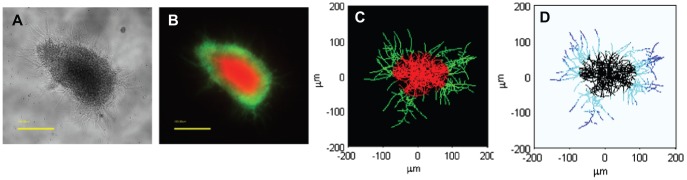
Microscopy images of *Streptomyces coelicolor* pellet compared to model simulations. A. Phase-contrast image of a pellet. **B.** Fluorescence image of the pellet using green SYTO9 (live cells) and red propidium iodide (likely dead cells) nucleic acid stains. **C.** Model simulation of a live/dead pellet at the end of the exponentially growing phase. Green colour corresponds to live cells with high consumption rate for oxygen 70% or more, and red colour illustrates the cells surrounded by less than ca 50% of the external oxygen present at the start of the incubation. **D.** The different metabolic states of the hyphae within the pellet at the end of the exponentially growing phase. Blue colour at the outskirts of the pellet marks the actively growing hyphae. Cyan colour corresponds to hyphae producing antibiotics. Black colour, at the core of the pellet, illustrates metabolically inactive hyphae with only maintenance demands for oxygen.

Now that we have a validated model framework, we use simulations to predict the cell and pellet heterogeneity in filamentous growth **(** **Fig. 7**). The simulation (**Fig. 7C**) indicates the switch from active to inactive hyphae at the interface of green/red areas of pellet. Part D in **Fig. 7** shows specific locations of the metabolic switch from primary to secondary metabolism where actively growing hyphae are predicted to be at the outskirts of the pellet (blue). More centrally are the parts of the hyphae that produce antibiotics (cyan). Within the simulation time scale no dead hyphae (light blue) are seen, instead at the core of the pellet the hyphae are still alive, however, they are not actively growing nor are they producing antibiotics, yet they still have maintenance requirements for oxygen (black).

To predict the external oxygen concentration surrounding cells located at the interface of live/dead stained areas, a number of simulations were run using different values of external oxygen concentration for the switch from green (live) to red (likely to be dead) areas and measuring the resulting red area. The red areas in simulations are then compared to the average red area seen in experimental data, where the average PI area were observed to be 1.71×10^4^ μm^2^ at the end of log phase. Using this comparison the model predicts that the shift seen in experiments from green to red occurs when external oxygen concentration drops to ca 50–55% (**Table** **5**).

**Table 5:** The average area of propidium iodide stained cells in experimental data (1.71×10^4^ μm^2^) is used to predict the oxygen concentration level at the interface of live/dead stained cells.

External O_2_ concentration^1^ at the interface of green/red areas in the simulations (%)	Average red (PI) area in simulations (×10^4^ μm^2^)
40%	0.62
45%	1.04
50%	1.53
51%	1.45
52%	1.56
53%	1.66
53.5%	1.78
54%	1.81
55%	1.89
60%	2.48
65%	2.89

1 Percentage scaled to the total carrying capacity of oxygen in media.

#### Network morphology

**Figure 8:**
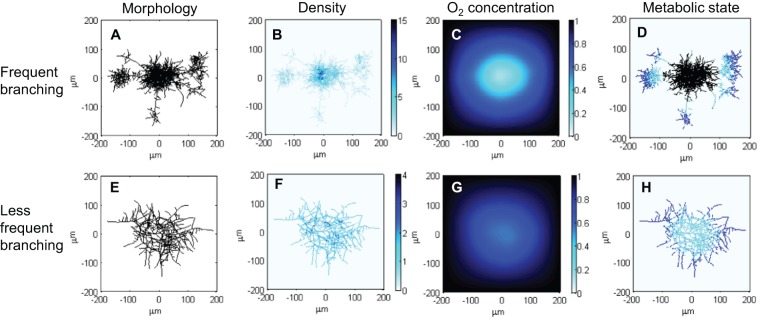
The effect of different branching patterns on pellet morphology, density, oxygen consumption and metabolism. In simulations **A–D** a frequent branching pattern using the previously measured apical and inter-branch distances of Jyothikumar et al. [36]. **E–H** illustrate less frequent branching pattern using the previously determined parameters of Allan and Prosser [37]. **B** and **F** show the hyphal density within a pellet and **C** and **G** show the rate of consumption of external oxygen. In **D** and **H** the metabolic state of hyphae is shown where blue colour corresponds to actively growing hyphae, cyan colour illustrates antibiotic producing hyphae, and black colour marks metabolically inactive hyphae with only maintenance demand for oxygen. The highest oxygen consumption is observed where the hyphae are densest. Less frequent branching produces larger, less dense pellets that demand less oxygen and are metabolically active across the whole pellet. See Table S1. for parameter values.

To find the effect that different branching patterns have on network morphology, oxygen consumption and metabolism, we ran simulations with varying parameters for apical (first branch point) and inter-branch distances as previously published for *S coelicolor* [36], [37] (see Table S1 for parameter values used). As observed in Fig. 8, increasing branching frequency in the model affects pellet morphology (**Fig. 8:** **A–H**). Interestingly, the model prediction of pellet morphology using frequent branching parameters shows the appearance of satellite pellets that are likely to break off from the original pellet under experimental conditions. A reassuring conclusion about the good fit between the model and the empirical work can be affirmed by observing the apparent that high hyphal density co-localises with increased branching frequency (**Fig. 8:** **B** and **F**). The oxygen concentration for both of the cases is shown in **Fig. 8:** **C and G**, where dense pellet areas have higher oxygen consumption. Images in **Fig. 8:** **D and H** show prediction of the metabolic state of hyphae suggesting that less frequent branching creates larger and less dense pellets that consume less oxygen and stay fully metabolically active.

#### Average hyphal velocity

**Figure 9:**
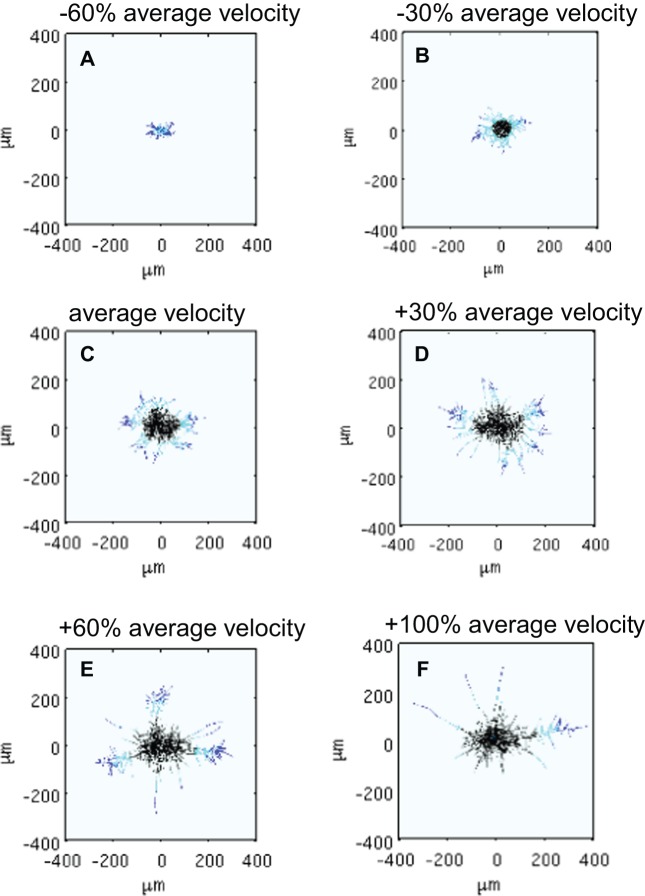
Increasing hyphal elongation velocity affects the network morphology. Simulation results show the shape of a network using an average tip elongation rate that is −60% (**A**), −30% (**B**), 0% (**C**)**,** +30% (**D**), +60% (**E**) and +100% (**F**) of the experimentally observed value (6.3 μm/h). Note that to accommodate the increased velocities, the domain size was increased to 500 μm×500 μm, 600 μm×600 μm, 800 μm×800 μm in (**D**), (**E**) and (**F**), respectively. Note: the images were manually adjusted to show 400 μm×400 μm coordinates to allow a comparison between the different cases.

In our model the hyphae elongate during the rapid growth phase according to an average velocity of 6.3 μm/h, based on experimental values. To determine the effect of different elongation rates and to test the effects of domain size on growth characteristics, we ran multiple simulations using average velocity values of 2.5 μm/h (−60%), 4.4 μm/h (−30%), 8.2 μm/h (+30%), 10 μm/h (+60%) and 12.6 μm/h (+100%), where the value in brackets corresponds to a percentage difference from our original assumed average velocity (see Table 1). In simulations, we observe that the decreased velocity results in smaller pellets with delayed antibiotic production. Increasing the velocity significantly affects overall morphology, with the pellet growing quicker with antibiotic producing cells appearing earlier. However, as the main body of the pellet stays approximately at the same size a few long, unbranched hyphae emerge from the main body forming sub-pellets (**Fig. 9**). This simulation outcome reveals two unexpected aspects of pellet development in our model. Firstly, within the simulation time the spatial domain needed to be increased such that the hyphal tips did not hit the simulated domain boundary as a result of rapid extension. Since oxygen has a limited diffusion distance, as the domain boundary is enlarged the oxygen distribution is affected. This artefact only affects the high velocity simulations where the oxygen does not diffuse through the space sufficiently such that the oxygen concentration at the pellet interior will be low. The lack of oxygen near the pellet surface makes it impossible for most of the hypha to continue elongating, and the long hypha seen in the simulations, are a result of the only few hyphal tips still reaching the area where adequate oxygen concentration for growth is present. Secondly, elongating hyphae appear unbranched even though the branching frequency is assumed to increase with increasing hyphal length. Our assumption in the model is that once the branching event occurs, the new branching point is calculated from the previous branching point. Since the previous branching point does not significantly change, the new branching point will emerge at the average interbranch distance of l_2_. Since this new position is already in the area of inadequate external oxygen concentration for growth, the branching event is most likely going to be unsuccessful. Therefore, it was noted that the model is favouring unsuccessful branching events when oxygen concentration is limited.

#### Insight into branching

**Figure 10:**
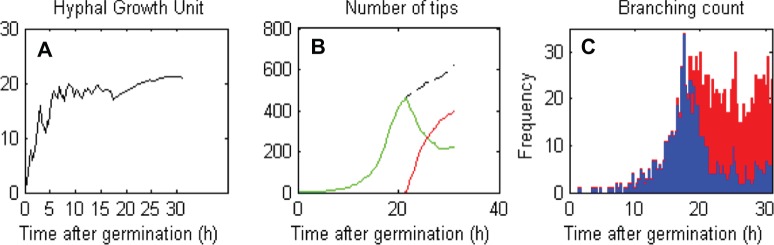
Additional insight into network characteristics. The model provides information on the hyphal growth unit (**A**), number of tips (**B**) and branching count (**C**) inside a dense network. In the simulation **B**, the black, dashed curve corresponds to the total number of tips, and green and red curves illustrate the total number of live and dead tips respectively. In **C**, the blue bars show the frequency of successful branching events and the red bars, the frequency of unsuccessful ones.

The model also allows us to gain quantitative insight into processes that are very difficult or impossible to obtain experimentally. Using the standard parameters for *Streptomyces* (see table 1), we can use the model to measure the hyphal growth unit (HGU) and the total number of tips inside a pellet (**Fig. 10:** **A & B**) – measurements that are almost impossible to make by microscopy. HGU is routinely used as a metric for growth of hyphal organisms. It is defined as total length of the hyphae divided by the number of tips and is a useful measure of the growth behaviour of a hyphal population. In our simulations the HGU is close to the HGU data previously measured by Allan and Prosser [37] where the HGU initially oscillated and subsequently reached a constant value. Similarly, the total numbers of tips in our simulations increase throughout the growth curve. The model also allows us to track the frequency of successful (blue) and unsuccessful (red) branching events during simulations (**Fig. 10** **C**). At the start of the growth curve, most of the branching events are successful with increasing frequency, with successful branching, declining rapidly following the onset of antibiotic production.

**Figure 11:**
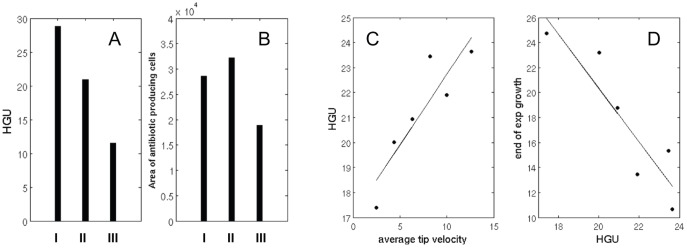
Model predictions using different branching patterns. Three different branching patterns for less frequent (I), standard (II) and frequent (III) branching affect the HGU (**A**) and the area of the antibiotic producing cells (**B**) at the end of log phase. Regression lines utilized to determine the HGU and the time of maximum succesful branching (**C** and **D** respectively) based on average tip velocities of 2.5 μm/h (−60%), 4.4 μm/h (−30%), 6.3 μm/h (±0), 8.2 μm/h (+30%), 10 μm/h (+60%) and 12.6 μm/h (+100%).

Using the data from the different branching pattern simulations (**Fig. 8**) we can draw further biological insight into the HGU and the hyphal area for antibiotic producing cells at the end of log phase (**Fig. 11** **A & B**). In Figure 11 the HGU (A) and area of antibiotic producing cells (B) for the apical and inter-branch lengths associated with the less frequent branching patterns (**I**) observed in the study of Allan and Prosser [37], (**III**) is for parameter values associated with the frequent branching study of Jyothikumar *et al*. [36] and (**II**) is for branch lengths as measured in this study at a 10 h time point of the growth curve experiment, in which we see a long apical branch distance and short inter-branch distance (which we will refer to as the standard parameters, see Table 1 for the model parameter values for this case). As expected, increasing branching frequency in the model reduces the HGU (**Fig. 11** **A**). This monotonic form, however, is not repeated in **Fig. 11** **B**, which shows that the area of antibiotic producing cells maximizes for standard parameters (**II**)**,** indicating that both apical and inter-branch lengths are crucial determinants of antibiotic production.

**Figure 12:**
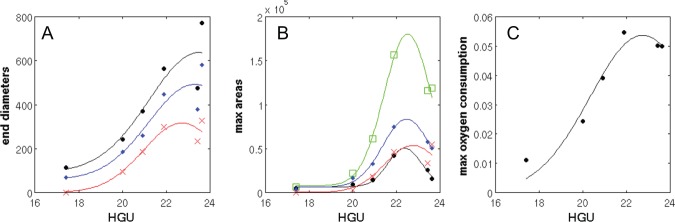
Model predictions using different average tip velocities. The relationships between end diameters and areas of the different metabolic regions using average tip velocities of 2.5 μm/h (−60%), 4.4 μm/h (−30%), 6.3 μm/h (±0), 8.2 μm/h (+30%), 10 μm/h (+60%) and 12.6 μm/h (+100%) versus HGU (**A** and **B**, respectively). The metabolic state of the network is indicated for actively growing cells (black circles), antibiotic producing cells (blue diamonds), maintenance requirements only cells (red crosses) and the total area of network (green rectangles). In (**C**), we plot the maximum rates of oxygen consumption in these simulations versus the calculated HGUs.

Data from the velocity simulations (**Fig. 9**) can be similarly used infer additional insight. For example, plotting HGU at the end of log phase versus average tip velocity (**Fig. 11** **C**) and the time of maximum successful branching versus these calculated HGUs (**Fig. 11** **D**) shows congruence, as would be expected when exponential growth is underway (i.e. fast extension rate, rapid branching). Again, using these calculated HGUs, we can also observe the relationships between end diameters and areas of the different metabolic regions versus HGU (see **Fig. 12** **A, B)**. Note that the different states of metabolism resemble growth curve characteristics with lag phase, exponential growth and stationary/death phases. We also plot the maximum rates of oxygen consumption in these simulations versus the calculated HGUs (see **Fig. 12** **C**). Not only can these plots be used for prediction purposes but also they highlight that an optimal value of average tip velocity for exponential growth exists and can be utilised in the design of industrial processes through the application of rational process design.

## Discussion

Branched networks are found throughout biology. The ability to achieve exponential network growth through branching has been adopted by many systems across a range of organisms either as a strategy [38], [39] or as an emergent property of apical growth [10], [40]. Here, we present a model that allows the visualisation of pellet development in *Streptomyces* and the estimation of difficult or impossible to obtain experimental measures, such as Hyphal Growth Units (HGU), pellet density, the prediction of metabolic status versus time across a pellet and the precise identification of antibiotic producing cells.

The model is able to predict the physiological heterogeneity within a pellet showing that the hypothesis of substrate-limitation within each pellet is significant in causing cessation of growth and cell death in the centre of pellets. The ability to test the model prediction through our use of BacLight fluorescent staining and also to compare the results to other studies of cell pellets [41] and to oxygen measurements [42] means that we can validate our modelling framework. Accurately predicting this behaviour from our model with verification through experimental testing allows further layers of complexity to be tested by the model. Antibiotic production (and other secondary metabolites) are often regulated by the availability of nutrients, and the ability of the model to predict those cells producing a product in an industrial setting will have great utility in assessing the morphology of pellets and the nutrient concentrations likely for optimised production. It is clear from the model that some of the inefficiency observed in fermentations may be the result of less than 50% of the biomass within a process producing the desired product, such that through manipulation of morphology, productivity can be enhanced. Several mutants are known to affect pellet formation, through fragmentation and also significantly enhance the production of a desired product [18], [19]. This should provide a rational framework for the rapid characterisation of cell division mutants that may be useful in an industrial process.

Interestingly, the number of tips in the model increases throughout, as would be expected, given that branching is a requirement for exponential growth in hyphal organisms. The model demonstrates that the number of successful branching events drops at the onset of antibiotic production, correlating with the decrease in growth rate that is observed in experiments at this time, indicating that our model can link the cessation of exponential growth and nutrient limitation to the production of antibiotics. The relatively few, easily measured parameters allow significant biological and functional information to be gathered from simulations.

To understand the hyphal elongation or pellet formation of filamentous organisms, several mathematical models have previously been constructed [14], [15], [26], [28]–[30], [42], [43]. Some of these models do take into account external substrate and metabolic state of cells, however only very few include antibiotic production. Our discrete model framework is able to predict the location of antibiotic producing cells in a pellet without having to compromise on the structure of a single hypha. Our model has the advantage that it is constructed from a few parameters that can be determined from experimental data. This tight link between mathematics and biology and the low number of unknown parameters makes the model very powerful. Thus ensures that we have a well-validated framework which we can use as a solid platform for further modelling applications. The model can be extended for future studies on biological processes such as the internal movement of proteins, DNA in hyphae and can be broadened to encompass any form of branched network structure. Additionally the information derived form these processes can be incorporated in to rational design of bioprocesses.

## Materials and Methods

### Bacterial strain and culturing

*Streptomyces coelicolor* strain M145 [44] was used in this study. The bacteria were routinely cultured and maintained following standard procedures [44]. Growth curve experiments were carried out at 30°C in Yeast Extract Malt Extract (YEME) media lacking sucrose [44] in two-litre Erlenmeyer flask without baffles or springs, shaking at 220 rpm. Cultures were inoculated with 1×10^5^ cfu/ml bacterial spores in 400 ml of media. Biomass concentration was determined in triplicate by vacuum-filtering 5 mL of culture onto pre-weighed, glass microfiber filters (GF/C, Whatman, UK). The filters and biomass were washed twice with 5 mL of distilled water and dried to a constant mass. The concentration of oxygen in media was determined using a dissolved oxygen probe (Mettler Toledo, UK).

### Microscopy and image analysis

Bacterial viability was estimated by microscopy using Live/dead® BacLight^TM^ bacterial viability kit (Molecular Probes, L7007, Invitrogen Detection Technologies, Leiden, The Netherlands). The maximum excitation/emission for SYTO9 and PI are 480/500 nm and 490/635 nm respectively. Microscopic slides were prepared in the dark by mixing 15 μl of culture with 15 μl of 0.0334 mM SYTO9 and 0.20 mM PI stain in distilled water. Images were observed using a Nikon TE2000S inverted fluorescence microscopy at x200. Images were captured using a Hamamatsu Orca-285 Firewire digital charge-coupled device camera. Exposure times were 20 ms for phase-contrast and fluorescence imaging throughout the growth curve analysis. For PI the initial exposure time was 50 ms for 12 h to 24 h time points and 20 ms for 37 h to 85 h time points. Pictures were analysed using IPLab scientific imaging software version 3.7 (Scanalytics, Inc., Rockville, USA) and an automated image analysis was performed for fluorescence images using iterative mode as per the software manufacturer's instructions. The resulting segmentation of the pellets was manually verified to cover the area of the pellet. Statistical analysis of the hyphal and pellet measurements was performed using Microsoft Office Excel software. Hyphal growth unit was calculated as the total length of hyphae divided by the total number of tips [13].

## Supporting Information

Table S1 Parameter values for frequent and less frequent branching patterns used in Fig. 8.
(DOC)
