# Supplementary material for: A Flexible Mathematical Model Platform for Studying Branching Networks: Experimentally Validated Using the Model Actinomycete, Streptomyces coelicolor
Source: PLoS One. 2013 Feb 18;8(2):e54316. doi: 10.1371/journal.pone.0054316 (PMC3575473; doi:10.1371/journal.pone.0054316)
Supplement: Table S1 — Parameter values for frequent and less frequent branching patterns used in Fig. 8. (DOC) [file pone.0054316.s001.doc]

**Supplementary Data Table S1.** Parameter values for frequent and less frequent branching patterns used in Fig. 8.

| **Frequent branching^1^** | | |
| --- | --- | --- |
| **Parameter** | **Symbol** | **Value** |
| Average apical length | *l_1_* | 10.94 μm |
| Average interbranch length | *l_2_* | 7.63 μm |
| Standard deviation of apical length | *l_1_sd* | 2.85 μm |
| Standard deviation of interbranch length | *l_2_sd* | 6.68 μm |
| **Less frequent branching^2^** | | |
| **Parameter** | **Symbol** | **Value** |
| Average apical length | *l_1_* | 35.2 μm |
| Average interbranch length | *l_2_* | 9.91 μm |
| Standard deviation of apical length | *l_1_sd* | 1.12 μm |
| Standard deviation of interbranch length | *l_2_sd* | 0.52 μm |

^1^ as previously measured by Jyothikumar *et al*. [1].

^2^ as previously measured by Allan and Prosser [2].

# References

1. Jyothikumar V, Tilley EJ, Wali R, Herron PR (2008) Time-lapse microscopy of Streptomyces coelicolor growth and sporulation. Applied and Environmental Microbiology 74: 6774–6781.

2. Allan EJ, Prosser JI (1983) Mycelial Growth and Branching of Streptomyces coelicolor A3(2) on Solid Medium. Microbiology 129: 2029–2036.
